# Prevalence and related factors of cognitive frailty in diabetic patients in China: a systematic review and meta-analysis

**DOI:** 10.3389/fpubh.2023.1249422

**Published:** 2023-10-19

**Authors:** Junjie Peng, Limei Ming, Jiaming Wu, Yunchuan Li, Shuhua Yang, Qin Liu

**Affiliations:** ^1^School of Nursing, Yunnan University of Chinese Medicine, Kunming, Yunnan, China; ^2^The First People's Hospital of Yunnan Province, Kunming, Yunnan, China; ^3^Postdoctoral Research Station of Public Administration, Yunnan University, Kunming, Yunnan, China

**Keywords:** cognitive frailty, diabetes, prevalence, relevant factors, meta-analysis

## Abstract

**Objective:**

Cognitive frailty (*CF*) is characterized by physical frailty and potentially reversible cognitive impairment without Alzheimer’s disease and other dementias. Clarifying the prevalence and related factors of cognitive frailty can help researchers understand its epidemiological status and formulate intervention measures. This study aims to conduct a systematic review and meta-analysis of the prevalence and related factors of *CF* in diabetic patients in Chinas to better understand the current status of *CF* in diabetic patients in China and develop effective intervention measures for related factors.

**Methods:**

PubMed, Web of Science, Embase, Cochrane Library, CNKI, Weipu(VIP), WANFANG, China Biology Medicine (CBM) and DUXIU were searched to collect epidemiological data on Chinese diabetic patients. Articles published through May 29, 2023, were searched. The number of diabetes with *CF* and the total number of diabetes in the included studies were extracted to estimate the prevalence of diabetes with *CF.* For factors related to diabetes with *CF*, odds ratios (*OR*) and 95% confidence intervals (*CI*) were used for estimation.

**Results:**

A total of 248 records were screened, of which 18 met the inclusion criteria. The results of meta-analysis showed that the prevalence of Chinese diabetic patients with *CF* was 25.8% (95% *CI* = 19.7 to 31.9%). Subgroup analysis showed that hospital prevalence was higher than in the community and in women than in men. Combined estimates showed that depression, malnutrition, advanced age (≥70, ≥80), combined chronic diseases ≥4 and glycated hemoglobin ≥8.5 were risk factors for *CF* in diabetics patients in China, with regular exercise and high education level (≥ college) as protective factors.

**Conclusion:**

Cognitive frailty was common in diabetic patients in China. Such populations should be screened early and intervened with relevant factors.

**Systematic review registration**: A systematic review of this study evaluated the registered websites as https://www.crd.york.ac.uk/PROSPERO/, CRD42023431396.

## 1. Introduction

Diabetes is a common chronic disease. The latest data from the International Diabetes Federation (IDF) in 2021 showed that the number of people with diabetes in the world was 537 million, which is expected to increase to 643 million by 2030 (1), and the related expenditure was US $966 billion, accounting for 11.5% of the total global health expenditure. The health problems of patients with diabetes deserve the attention of medical and health professionals in all countries. Population aging will increase the expected prevalence of diabetes by 16%. As the country with the largest number of diabetes patients, China will face certain medical pressure, and the health problems of diabetes patients will be more prominent.

Physical frailty and cognitive impairment are common symptoms in the older adults (2). Frailty refers to a physiological syndrome in which multiple systems of the body are dysfunctional, resulting in decreased physiological reserve and increased susceptibility to stimulation (3). Cognitive impairment is considered to be the early stage of dementia, and the lack of cognitive ability will lead to the impairment of daily function of patients (4). With the progress of research, more studies have found that there is a certain correlation between physical frailty and cognitive impairment (5). Therefore, the concept of *CF* has been proposed. The concept of *CF* was first proposed in 2013 (6) and is defined as the coexistence of physical frailty and cognitive impairment in the absence of other neurological diseases such as Alzheimer’s disease. Ruan et al. proposed two subtypes of *CF* in 2015 (7): reversible *CF*, which refers to the existence of subjective cognitive decline and/or related markers on the basis of physical frailty; and potentially reversible *CF* refers to physical frailty accompanied by mild cognitive impairment. The concept of *CF* is more conducive to the transformation of the assessment method of the older adults from single-dimensional to multidimensional and the maintenance of the physical and mental health of older adults individuals.

In the mechanism of *CF*, there is a bidirectional relationship between physical frailty and cognitive impairment, which affect and promote each other, forming a vicious circle of decline in physical function and cognitive function of the older adults (8, 9). On this basis, hypoglycemic events and sarcopenia in diabetic patients can be used as facilitating and linking links in this process. Hypoglycemic events: Abdelhafiz et al. (10) suggested a bidirectional relationship between hypoglycemia and physical frailty and cognitive impairment. In terms of cognitive function, hypoglycemia events can increase the risk of cognitive dysfunction in patients, and cognitive dysfunction may reduce self-care and rational drug use and increase the risk of blood glucose fluctuation. In terms of physical frailty, diabetic patients with frequent hypoglycemia are often complicated with diseases, malnutrition, weight loss and have potential physical frailty. Diabetic patients with physical frailty have a higher risk of hypoglycemia due to reduced physiological reserve. Sarcopenia: Hormone deficiency (11), neuropathy (12) and chronic inflammation (13) caused by diabetes can affect the growth and metabolism of skeletal muscle cells, reduce muscle mass and delay muscle regeneration, leading to an increased risk of sarcopenia. Sarcopenia can also lead to an increased risk of physical frailty and cognitive impairment in patients (14, 15).

*CF* can not only predict adverse health outcomes in the older adults but also increase the risk of adverse health outcomes. A 7-year follow-up study found that older adults people with *CF* had increased cumulative length of hospital stay compared with healthy older adults people (*OR* = 1.48) (16). A study of older adults in an Italian community showed that people with *CF* had more severe physical activity impairments than healthy older adults (17). Rivan et al. (18) showed that *CF* was not only an influencing factor for falls in the older adults (*OR* = 2.98, 95% *CI*: 1.78–4.99) but also significantly predicted the incidence of disability (*OR* = 5.17, 95% *CI*: 1.11–24.21). The results of another meta-analysis showed that *CF* may be an early sign of the onset of dementia and is an important predictor of dementia (19). Feng et al. used a 3-year prospective cohort study and concluded that *CF* significantly increased the risk of death in the older adults (OR = 5.12, 95% CI: 3.00–8.74) (20). However, *CF* is reversible (7). Early screening and intervention of diabetic *CF* patients will help patients restore normal cognitive function, maintain self-care ability, delay or prevent dementia, and reduce the occurrence of adverse health events.

Since the concept of *CF* was systematically proposed in 2013, the number of related studies has been increasing, while the research progress in China is relatively backward. Although many epidemiological studies have been conducted to further understand the prevalence and associated factors of *CF*, there are currently insufficient studies on the prevalence and associated factors of *CF* in diabetic patients. To improve the attention of Chinese medical workers and facilitate the promotion of related research progress, it is necessary to conduct a meta-analysis on the current epidemiological status of *CF* in diabetic patients in China. Based on the reversibility of *CF*, clarifying the related factors between diabetic patients and *CF* will help to carry out relevant intervention research to improve the coping ability of this group to maintain their own health.

Many epidemiological studies have been conducted in China to further understand the prevalence and related factors of *CF*, but there are still insufficient studies on the prevalence and related factors of *CF* in diabetic patients. Therefore, this study aims to systematically evaluate the current status of *CF* in diabetic patients in China and analyze the prevalence of *CF* in diabetic patients by gender, age, region and education level to provide evidence for the development of health intervention measures and the government to formulate relevant public health strategies.

## 2. Method

This systematic review and meta-analysis was conducted according to the Preferred Reporting Item (PRISMA) updated guidelines for systematic reviews and meta-analyses (21). The protocol has been registered in the International Prospective Register of Systematic Reviews (PROSPERO) and the registration number is CRD42023431396.

### 2.1. Search strategy

PubMed, Web of Science, Cochrane Library, Embase, CNKI, Wanfang (VIP), China Biology Medicine disc (CBM), and DUXIU were searched for articles published up to May 29, 2023. Because *CF* topics are relatively new in the scientific literature, no time limits were used. The search was conducted using Medical Subject Headings (MeSH) and free terms, including Diabetes Mellitus, Diabete*, Cognitive Frailty, frailty, Cognitive Impairment. In addition, the search was extended by handsearching the reference lists of relevant studies, including reviews and included studies. The search strategy is shown in Table 1.

**Table 1:** Search strategies and terms.

Literature Library	Search queries	Search results
PubMed	#1 (((((((((China[MeSH Terms]) OR (China[Title/Abstract])) OR (taiwan[MeSH Terms])) OR (taiwan[Title/Abstract])) OR (hongkong[MeSH Terms])) OR (hongkong[Title/Abstract])) OR (Macau[MeSH Terms])) OR (Macau[Title/Abstract])) OR (Chinese*[Title/Abstract])) OR (china*[Title/Abstract])	625,800
	#2 (((Diabetes Mellitus[MeSH Terms]) OR (Diabetes Mellitus[Title/Abstract])) OR (Diabete*[Title/Abstract])) OR (diabetic*[Title/Abstract])	833,544
	#3 Cognitive frailty[Title/Abstract]	313
	#4 (frailty[Title/Abstract]) AND (Cognitive Impairment[Title/Abstract])	1,469
	#5 (((((Risk factors[MeSH Terms]) OR (Risk factors[Title/Abstract])) OR (Prevalence[MeSH Terms])) OR (Prevalence[Title/Abstract])) OR (Related factor*[Title/Abstract])) OR (associated factor*[Title/Abstract])	1,985,809
	#6 = #3 OR #4	1,594
	#7 = #1 AND #2 AND #5 AND #6	13
web of science	#1 (((((TS = (China)) OR TS = (taiwan)) OR TS = (hongkong)) OR TS = (Macau)) OR AB = (Chinese*)) OR AB = (china*)	1,699,850
	#2 ((TS = (Diabetes Mellitus)) OR AB = (Diabete*)) OR AB = (diabetic*)	1,143,361
	3 AB = (Cognitive frailty)	3,565
	#4 (AB = (frailty)) AND AB = (Cognitive Impairment)	1,685
	#5 (((TS = (Risk factors)) OR TS = (Prevalence)) OR AB = (Related factor*)) OR AB = (associated factor*)	5,108,992
	#6 #3 OR #4	3,565
	#7 #1 AND #2 AND #5 AND #6	19
EMBASE	#1 china:ti,ab,kw OR taiwan:ti,ab,kw OR ‘hong kong’:ti,ab,kw OR macau:ti,ab,kw OR chinese*:ti,ab,kw OR china*:ti,ab,kw	682,029
	#2 ‘diabetes mellitus’:ti,ab,kw OR diabete*:ti,ab,kw OR diabetic*:ti,ab,kw	1,184,989
	#3 ‘cognitive frailty’:ti,ab,kw	387
	#4 frailty:ti,ab,kw AND ‘cognitive impairment’:ti,ab,kw	2,568
	#5 ‘risk factors’:ti,ab,kw OR prevalence:ti,ab,kw OR ‘related factor*’:ti,ab,kw OR ‘associated factor*’:ti,ab,kw	1,905,808
	#6 #3 OR #4	2,721
	#7 #1 AND #2 AND #5 AND #6	13
Cochrane Library	#1 (China):ti,ab,kw OR (taiwan):ti,ab,kw OR (hongkong):ti,ab,kw OR (Macau):ti,ab,kw OR (Chinese*):ti,ab,kw	51,315
	#2 (Diabetes Mellitus):ti,ab,kw OR (Diabete):ti,ab,kw OR (diabetic*):ti,ab,kW	113,440
	#3 (Cognitive frailty):ti,ab,kw	779
	#4 (frailty):ti,ab,kw AND (Cognitive Impairment):ti,ab,kw	284
	#5 (Risk factors):ti,ab,kw OR (Prevalence):ti,ab,kw OR (Related factor*):ti,ab,kw OR (associated factor*):ti,ab,kw	231,740
	#6 = #3 OR #4	779
	#7 = #1 AND #2 AND #5 AND #6	6
CNKI	((((Topic% = ‘认知衰弱’ or Title% = ‘认知衰弱’) OR (Legacy Topic = ‘认知障碍 and 衰弱’)) AND (Topic% = ‘糖尿病’ or Title% = ‘糖尿病’)) AND ((((((Legacy Topic = ‘影响因素’) OR (Legacy Topic = ‘危险因素’)) OR (Legacy Topic = ‘相关因素’)) OR (Legacy Topic = ‘预测因素’)) OR (Legacy Topic = ‘患病率’)) OR (Legacy Topic = ‘流行病学’)))	38
VIP	(M = 糖尿病 OR R = 糖尿病) AND ((M = 认知衰弱 OR R = 认知衰弱) OR ((M = 认知障碍 OR R = 认知障碍) AND (M = 衰弱 OR R = 衰弱))) AND (R = 影响因素 OR R = 危险因素 OR R = 相关因素 OR R = 预测因素 OR R = 患病率 OR R = 流行病学)	36
WANFANG	Topic:(糖尿病) and (Topic:(认知衰弱) or (Abstract:(认知障碍) and Abstract:(衰弱))) and (Abstract:(影响因素) or Abstract:(危险因素) or Abstract:(相关因素) or Abstract:(预测因素) or Abstract:(患病率) or Abstract:(流行病学))	64
CBM	#1 “糖尿病”[Title] OR “糖尿病”[Abstract:] OR “糖尿病”[keyword]	438,489
	#2 “认知衰弱”[Title] OR “认知衰弱”[Abstract] OR “认知衰弱”[keyword]	114
	#3 “认知障碍”[Abstract] AND “衰弱”[Abstract]	118
	#4 “影响因素”[Abstract] OR “危险因素”[Abstract] OR “相关因素”[Abstract] OR “预测因素”[Abstract] OR “患病率”[Abstract] OR “流行病学”[Abstract]	585,143
	#5 (#3) OR (#2)	214
	#6 (#5) AND (#4) AND (#1)	19
DUXIU	(T = 糖尿病 | K = 糖尿病 | S = 糖尿病) * ((T = 认知衰弱 | K = 认知衰弱 | S = 认知衰弱) | (S = 认知障碍 * S = 衰弱)) * (S = 影响因素 | S = 危险因素 | S = 相关因素 | S = 预测因 | S = 患病率 | S = 流行病学)	36

### 2.2. Inclusion and exclusion criteria

Inclusion criteria were based on the PICO process: (1) The patient was diagnosed with diabetes; (2) Cross-sectional, case–control, or cohort studies; (3) There are clear diagnostic criteria for *CF*, which are both physical frailty and cognitive dysfunction, and other neuropathic diseases such as Alzheimer’s disease are excluded; (4) The article provides the prevalence of *CF* in diabetic patients or provides corresponding data that can calculate the prevalence; (5) Research is carried out in China.

The exclusion criteria: (1) duplicate publications; (2) data on outcome indicators could not be extracted; (3) review articles, case reports and letters to the editor; (4) documentation from the same region in the same year.

### 2.3. Literature screening and data extraction

Import records identified in databases and manual retrievals into Endnote X9.0. Based on the inclusion and exclusion criteria, two review authors independently assessed and screened the titles and abstracts recorded in Endnote X9.0 for inclusion. If there was disagreement, we consulted and discussed or listened to the opinions of third-party evidence-based medicine experts. Data from the included studies were extracted by the same two reviewers using a data extraction table in Excel, including author, year of publication, country, study design, sample size, participant characteristics (age and sex), *CF* assessment tool, prevalence (numerator and denominator), and relevant factors.

### 2.4. Qualitative evaluation

Literature quality assessment was conducted by two review authors according to the criteria, and disagreements were resolved by consultation or discussion with a third investigator. The cross-sectional study used the evaluation criteria recommended by the Agency (22), and the AHRQ criteria included 11 items with “yes,” “no” or “unclear.” The cohort study adopted the NOS evaluation criteria of the cohort study (23), and the NOS evaluated the literature through the method of three blocks with a total of 8 items. The evaluation of the quality of the literature adopted the semiquantitative principle of the star system, with a maximum score of 9 stars. The quality of each study was classified as poor, fair, or good.

### 2.5. Statistical analysis

EndNote X9 software was used for file management, an Excel table was used to extract data, and Stata 15.0 was used for analysis. The primary outcomes were the pooled prevalence and 95% confidence interval (*CI*) of *CF* in people with diabetes in China, and the factors associated with *CF* were estimated using the pooled odds ratio (*OR*) and 95% *CI*. Cochran’s *Q* and *I^2^* statistics were used to examine statistical heterogeneity between studies. If the test for heterogeneity detected a statistically significant difference (*I^2^* > 50%), we used a random-effects model; otherwise, we used a fixed-effect model. Begg’s test, Egger’s test, and funnel plots were used to assess publication bias. The significance level of the tests was considered less than 0.05.

## 3. Results

### 3.1. Literature screening process and results

**Figure 1:**
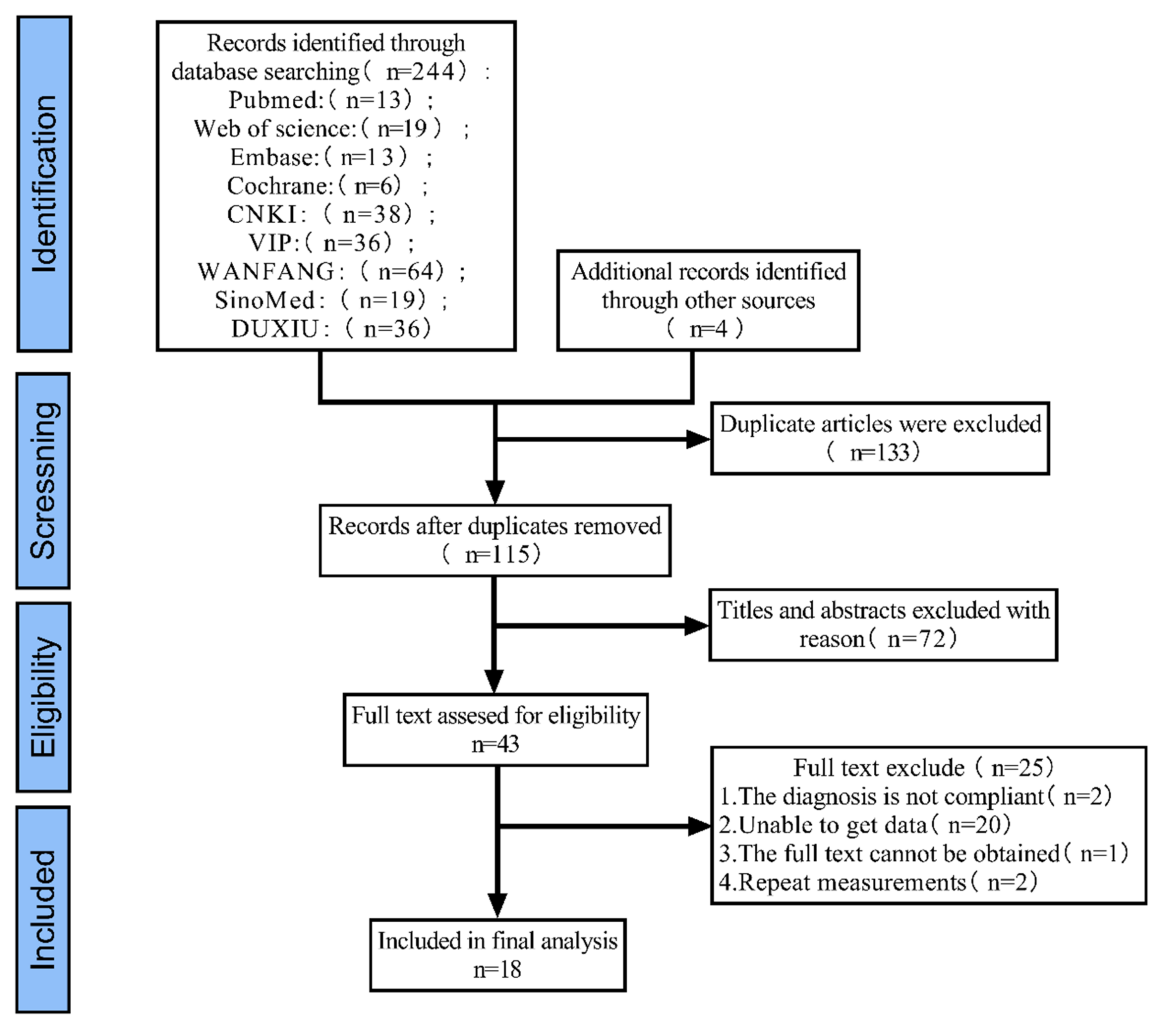
Flow chart of literature screening.

The results are shown in Figure 1, where a database search identified 248 records (the number of studies retrieved was small, which may be related to the fact that there are few studies on *CF* in diabetic patients in China), of which 133 were duplicates. By reading the title and abstract, 72 studies were eliminated and 43 studies were retained for full-text reading to assess eligibility. Ultimately, 18 studies were included.

### 3.2. Characteristics and quality evaluation results of the included literature

The characteristics of the study are shown in Table 2. A total of 18 studies included 4,030 Chinese people with diabetes, 16 studies had a cross-sectional design, and two studies had a cohort design. Sample sizes ranged from 81 to 395. In the areas where the survey was conducted, there were 8 in the south and 10 in the north. In terms of sample sources, there were 12 hospital sources and 6 community sources. In the diagnosis of *CF*, the evaluation tools of physical frailty were mainly FS and FP, with 7 and 8, respectively, and others such as SHARE-FI, CGA-FI and AWGS accounted for one each. Four of the cognitive function assessment tools used two different evaluation tools, and the rest were single evaluation tools, 8 studies used MMSE, 8 used MoCA, 3 studies used CDR, and one each used SCD-Q9, HDS-R and SPMSQ. The most commonly used combinations by researchers are FS or FP, MMSE or MoCA.

**Table 2:** Characteristics of included studies.

Author, year	Investigation time	Region	Sample source	Average age	Sample size	Number of checkouts	Male and female	Cognitive frailty assessment	
								Frailty	Cognitive impairment
Li et al. (24), 2022	August to December 2020	Hangzhou, Zhejiang	Community	≥60	252	22	148/104	FS	MMSE
Jiang et al. (25), 2022	March 2019 to March 2022	Beijing	Hospital	68.02 ± 6.41	350	30	168/182	FP	MMSE
Wang (26), 2022	March to October 2021	Shenyang, Liaoning	Hospital	67 (63, 72) (≥60)	300	65	152/148	FS	MMSE
Liu (27), 2021	April to December 2019	Urumqi, Xinjiang	Hospital	75.3 ± 6.8	395	82	166/225	FP	MoCA
Zhang et al. (28), 2023	January to December 2021	Hangzhou, Zhejiang	Hospital	≥60	233	48	114/109	FP	MMSE
Yang (29), 2022	March to October 2021	Nanning, Guangxi	Hospital	70.64 ± 7.43	225	76	107/118	SHARE-FI	MoCA
Fu and Zhao (30), 2023	March to September 2022	Dalian, Liaoning	Hospital	≥60	226	47	120/106	FS	MoCA
Wang (31), 2021	February 2019 to January 2021	Jinan, Shandong	Hospital	62.99 ± 6.65	186	105	109/77	FP	CDR, MoCA
Zhang et al. (32), 2020	January to June 2019	Zhengzhou, Henan	Community	69 ± 6.62	225	28	91/164	FP	MoCA, CDR
Han (33), 2021	August to December 2020	Shenyang, Liaoning	Community	67.81 ± 6.45	200	66	117/83	FS	SCD-Q9, MoCA
Yang (34), 2022	January 2021 to January 2022	Hefei, Anhui	Hospital	71.02 ± 7.36	252	114	132/120	FS	MMSE, MoCA
Kong et al. (35), 2020	June to October 2019	Xianning, Hubei	Community	≥65	291	25	/	FP	MMSE
Wang (36), 2021	November 2019 to September 2020	Tangshan, Hebei	Hospital	≥65	81	31	/	FS	CDR
Ma et al. (37), 2020	June 2017 to January 2019	Shenyang, Liaoning	Hospital	65	200	36	/	FS	MMSE
Chen (38), 2020	December 2018 to September 2019	Zhengzhou, Henan	Hospital	60	82	57	/	FP	MoCA
Wang et al. (39), 2019	November 2015 to January 2018	Chengdu, Sichuan	Hospital	60	238	59	/	CGA-FI	MMSE
Li et al. (40), 2019	November to December 2014	Jiangsu Rugao	Community	70	121	18	/	FP	HDS-R
Lee et al. (41), 2018	2006	Taiwan, China	Community	53 ~ 58	173	30	/	AWGS	SPMSQ

FS, FRAIL scale; MMSE, Mini-Mental State Examination; FP, Frailty Phenotype; MoCA, Montreal Cognitive Assessment; SHARE-FI, Shared Frailty Screening Tool; CDR, Clinical Dementia Rating; SCD-Q9, Subjective Cognitive Decline Questionnaire 9; CGA-FI, Frailty Index based on a Comprehensive Geriatric Assessment; HDS-R, Hasegama’s dementia scale; AWGS, Asian Working Group on Sarcopenia criteria; SPMSQ, Short Portable Mental Status Questionnaire.

### 3.3. Quality appraisal

Table 3 lists the assessment of methodological quality of all 18 studies included in this review. Of the cross-sectional studies, four studies were judged to be ‘good’ and 12 to be of ‘fair’ quality. Of the cohort studies, one was considered ‘good’ and one was classified as ‘fair’.

**Table 3:** Quality appraisal.

Cross-sectional studies(AHRQ)											
Author, year	1	2	3	4	5	6	7	8	9	10	11
Li et al. (24), 2022	Yes	Yes	Yes	Yes	No	Yes	Yes	Yes	No	Yes	No
Jiang et al. (25), 2022	Yes	Yes	Yes	Yes	No	Yes	No	Yes	No	No	No
Wang (26), 2022	Yes	Yes	Yes	Yes	No	Yes	Yes	Yes	Yes	Yes	No
Liu (27), 2021	Yes	Yes	Yes	Yes	No	Yes	Yes	Yes	No	No	No
Zhang et al. (28), 2023	Yes	Yes	Yes	Yes	No	Yes	No	Yes	No	No	No
Yang (29), 2022	Yes	Yes	Yes	Yes	No	Yes	Yes	Yes	Yes	Yes	No
Fu and Zhao (30), 2023	Yes	Yes	Yes	Yes	No	Yes	Yes	Yes	No	No	No
Wang (31), 2021	Yes	Yes	Yes	Yes	No	Yes	Yes	Yes	No	No	No
Zhang et al. (32), 2020	Yes	Yes	Yes	Yes	No	Yes	Yes	Yes	No	No	No
Han (33), 2021	Yes	Yes	Yes	Yes	No	Yes	No	Yes	No	No	No
Yang (34), 2022	Yes	Yes	Yes	Yes	No	Yes	Yes	Yes	No	No	No
Kong et al. (35), 2020	Yes	Yes	Yes	Yes	No	Yes	Yes	Yes	No	No	No
Wang (36), 2021	Yes	Yes	Yes	Yes	No	Yes	No	Yes	No	No	No
Ma et al. (37), 2020	Yes	Yes	Yes	Yes	No	Yes	No	Yes	No	No	No
Chen (38), 2020	Yes	Yes	Yes	Yes	No	Yes	Yes	Yes	Yes	Yes	No
Wang et al. (39), 2019	Yes	Yes	Yes	Yes	No	Yes	Yes	Yes	No	No	No

Cohort studies (NOS)								
Author, year	1	2	3	4	5	6	7	8
Li et al. (40), 2019	1	1	1	1	1	1	0	0
Lee et al. (41), 2018	1	1	1	1	0	1	1	1

### 3.4. Meta-analysis

**Figure 2:**
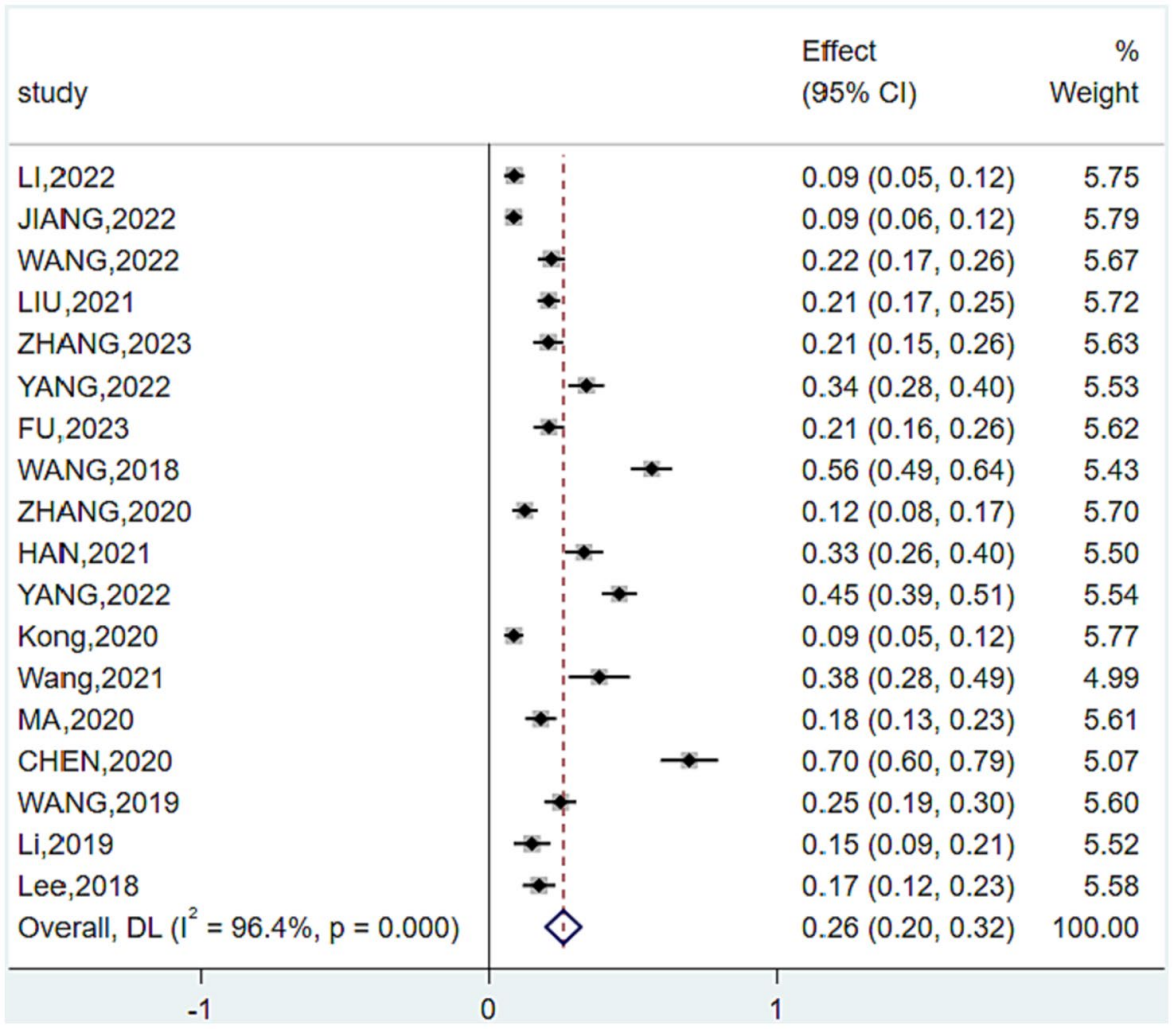
Forest plot of total prevalence.

Meta-analysis showed some heterogeneity in the included studies (*I^2^* = 96.4%, *p* < 0.001), so a random-effects model was used and subgroup analyses were performed to analyze sources of heterogeneity. The forest chart is shown in Figure 2, and the analysis results are shown in Table 4.

**Table 4:** Analysis of *CF* prevalence in patients with diabetes in China.

Outcomes	Included studies	Heterogeneity test results		Meta-analysis results	
		I2	*p* value	Prevalence	(95%*CI*)
Cognitive frailty	18	96.40%	<0.001	0.258	19.7–31.9%
Sample source					
Hospital	12	96.70%	<0.001	0.311	22.6–39.6%
Community	6	90.30%	<0.001	0.258	19.7–31.9%
Sex					
Male	11	94.60%	<0.001	0.232	15.1–31.3%
Female	11	94.50%	<0.001	0.267	18.3–35.1%
Hospital male	8	94.50%	<0.001	0.267	1.66–3.69%
Hospital female	8	94.30%	<0.001	0.295	4.11–5.89%
Community male	3	92.30%	<0.001	0.139	0.24–2.55%
Community female	3	94.30%	<0.001	0.192	0.42–3.42%
Region					
North	10	96.90%	<0.001	0.294	2.01–3.87%
South	8	95.80%	<0.001	0.216	1.32–3.00%
North China hospital	8	97.40%	<0.001	0.312	1.99–4.25%
South China hospital	4	92.70%	<0.001	0.310	2.04–4.16%
Age					
≥60	16	95.80%	<0.001	0.244	1.85–3.03%
60 ~ 69	7	88.50%	<0.001	0.147	0.90–2.05%
70 ~ 79	8	91.30%	<0.001	0.322	1.99–4.45%
≥80	8	85.80%	<0.001	0.573	4.04–7.43%
Educational level					
≤Primary school	9	95.50%	<0.001	0.445	2.76–6.13%
Junior high school	8	72.00%	0.001	0.272	1.77–3.68%
High school	8	69.10%	0.002	0.218	1.13–3.24%
≥College	9	0.00%	0.472	0.069	0.15–1.22%
Course					
1 ~ 9	7	96.60%	<0.001	0.261	1.37–3.86%
≥10	8	90.40%	<0.001	0.362	2.29–4.94%
Coexisting chronic conditions					
0 ~ 1	6	96.90%	<0.001	0.217	0.82–3.51%
≥2	6	87.60%	<0.001	0.315	1.86–4.43%
Depression	9	95.50%	<0.001	0.414	2.55–5.72%
Malnutrition	6	92.90%	<0.001	0.39	2.39–5.40%

Overall prevalence of *CF* in patients with diabetes in China. A total of 4,030 older adults diabetic patients in China were included in the 18 studies of this study, and 939 diabetic patients with *CF* were detected. The prevalence of the included studies was 8.60–56.50%. The total number of patients with diabetes and the number of *CF* patients were compared, and the overall prevalence of *CF* in Chinese patients with diabetes was 25.8% (95% *CI* (19.7 to 31.9%)).

Prevalence by sex. A total of 11 studies reported the prevalence of *CF* in both men and women, men and women prevalence was 23.2% (95% *CI* (15.1 to 31.3%)) and 26.7% (95% *CI* (18.3 to 35.1%)); 8 study carried out in the hospital, the hospital male and female *CF* prevalence was 26.7% (95% *CI* (1.66 to 3.69%)) and 29.5% (95% *CI* (4.11 to 5.89%)); 3 study in community, *CF* men and women with diabetes prevalence was 13.9% (95% *CI* (0.24 to 2.55%)) and 19.2% (95% *CI* (0.42 to 3.42%)).

#### 3.4.1. Prevalence by region

Two studies were included in hospitals and 6 were included in the community. The prevalence was 31.1% (95% *CI* (22.6 to 39.6%)) in hospitals and 25.8% (95% *CI* (19.7 to 31.9%)) in the community. There were 8 studies in the south and 10 studies in the north, and the prevalence was 29.4% (95% *CI* (2.01–3.87%)) and 21.6% (95% *CI* (1.32–3.00%)) in the north and south, respectively. According to hospital location, the prevalence was 31.2% (95% *CI* (1.99% CI 1.99 to 4.25%)) in northern hospitals and 31.0% (95% *CI* (2.04–4.16%)) in southern hospitals.

#### 3.4.2. Prevalence by age

There were 7 studies involving patients aged 60 ~ 69 years with a prevalence of 14.7% (95% *CI* (0.90–2.05%)), 8 studies involving patients aged 70 ~ 79 years with a prevalence of 32.2% (95% *CI* (1.99–4.45%)), 8 studies involving patients ≥80 years with a prevalence of 57.3% (95% *CI* (1.13–3.24%)), and a total of 16 studies involving older adults patients aged 60 years ≥ with a prevalence of 24.4%(95% *CI*(1.85–3.03%)).

#### 3.4.3. Prevalence by educational level

A total of 9 studies included patients with a primary school education and below, with a prevalence of 44.5% (95% *CI* (2.76 to 6.13%)), 8 included patients with a junior high school degree, with a prevalence of 27.2% (95% *CI* (1.77 to 3.68%)), 8 included patients with a high school education, with a prevalence of 21.8% (95% *CI* (1.99 to 4.45%)), and 9 studies included patients with a college education or above, with a prevalence of 0.69% (95% *CI* (0.15 to 1.22%)).

According to the course. A total of 7 studies included *CF* in diabetic patients with a course of 1 ~ 9 years, with a prevalence of 26.1% (95% *CI* (1.37–3.86%)), and a total of 8 studies contained data on diabetic patients with a course of ≥10 years, with a prevalence of 36.2% (95% *CI* (2.29–4.94%)).

According to the stratified number of combined chronic diseases. A total of 6 studies included data on diabetes patients with 0 ~ 1 chronic diseases, with a prevalence of 21.7% (95% *CI* (0.82–3.51%)), and a total of 6 studies included data on diabetic patients with ≥2 combined chronic diseases, with a prevalence of 31.5% (95% *CI* (1.86–4.43%)).

Analysis results of other subgroups. A total of 9 studies included data on diabetic patients with depression, with a prevalence of 41.4% (95% *CI* (2.55–5.72%)), and 6 studies included data on diabetic patients with malnutrition, with a prevalence of 39.0% (95% *CI* (2.39–5.40%)).

### 3.5. Analysis of *CF* related factors in diabetic patients in China

The analysis results of *CF* related factors in diabetic patients in China are shown in Table 5. The combined estimates showed that depression (*OR* = 1.117, 95% *CI* = 0.693 ~ 1.541, *p* < 0.001), malnutrition (*OR* = 1.420, 95% *CI* = 0.763 ~ 2.0771, *p* < 0.001), age (≥70) (*OR* = 0.717, 95% *CI* = 0.424 ~ 1.009, p < 0.001), age (≥80) (*OR* = 1.974, 95% *CI* = 0.910 ~ 3.037, *p* < 0.001), chronic disease≥4 (*OR* = 3.572, 95% *CI* = 1.910 ~ 5.233, *p* < 0.001) and glycated hemoglobin ≥8.5 (*OR* = 1.177, 95% *CI* = 0.500 ~ 1.8543, *p* = 0.001) were risk factors for *CF.* Education level (college or above) (*OR* = -1.245, 95% *CI* = -1.748 ~ −0.743, *p* < 0.001) and regular movement (*OR* = -1.330, 95% *CI* = -1.968 ~ −0.6919, *p* < 0.001) were protective factors. The pooled data showed that there was no significant difference in the association between education level (high school and above) (*p* > 0.05).

**Table 5:** Analysis of relevant factors.

Relevant factors	Number of studies	*OR* (95%*CI*)	*p* value	Heterogeneity		
				*Q* test	*p* value	*I^2^*
Depression	8	1.117 (0.693 ~ 1.541)	<0.001	17.54	0.014	60.10%
Malnutrition	6	1.420 (0.763 ~ 2.077)	<0.001	25.55	<0.001	80.40%
≥70	3	0.717 (0.424 ~ 1.009)	<0.001	1.98	0.371	0.00%
≥80	3	1.974 (0.910 ~ 3.037)	<0.001	5.94	0.051	66.30%
Regular exercise	5	-1.330 (−1.968 ~ −0.691)	<0.001	8.03	0.090	50.20%
≥High school	2	−0.900 (−1.949 ~ 0.149)	0.093	2.65	0.104	62.20%
≥College	2	−1.245 (−1.748 ~ −0.743)	<0.001	0	0.996	0.00%
Chronic disease≥4	2	3.572 (1.910 ~ 5.233)	<0.001	0.02	0.879	0.00%
Glycated hemoglobin≥8.5	2	1.177 (0.500 ~ 1.854)	0.001	0.29	0.593	0.00%

### 3.6. Sensitivity analyses and publication bias

**Figure 3:**
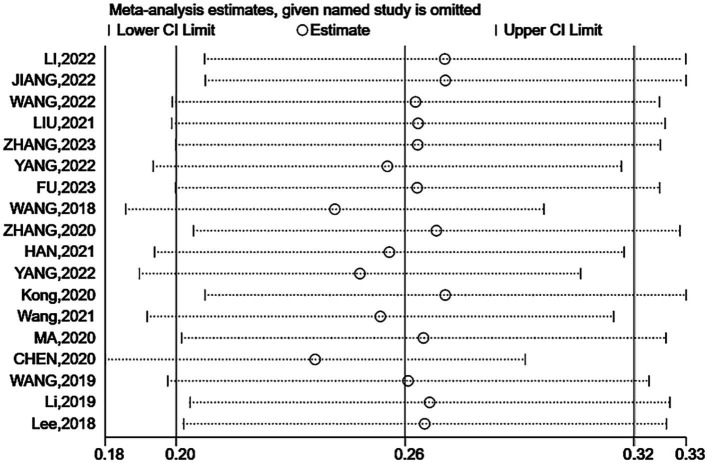
Sensitivity analysis.

**Figure 4:**
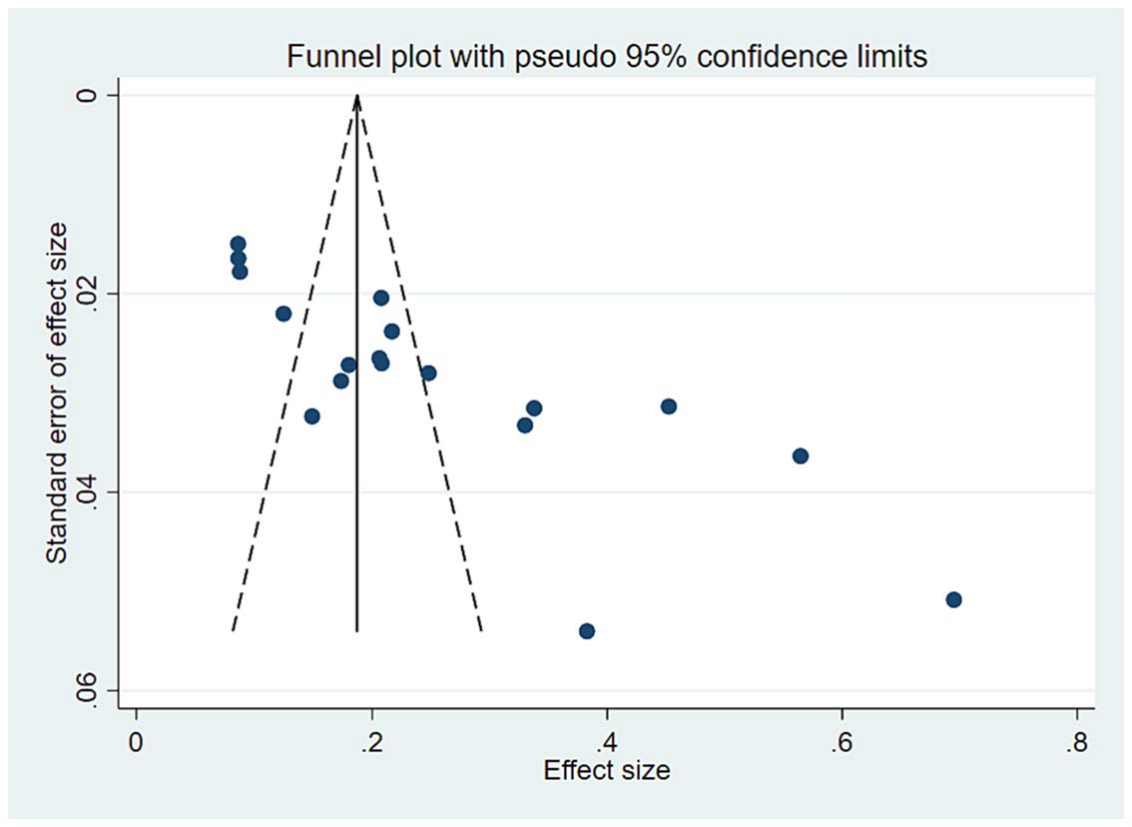
Funnel diagram.

**Figure 5:**
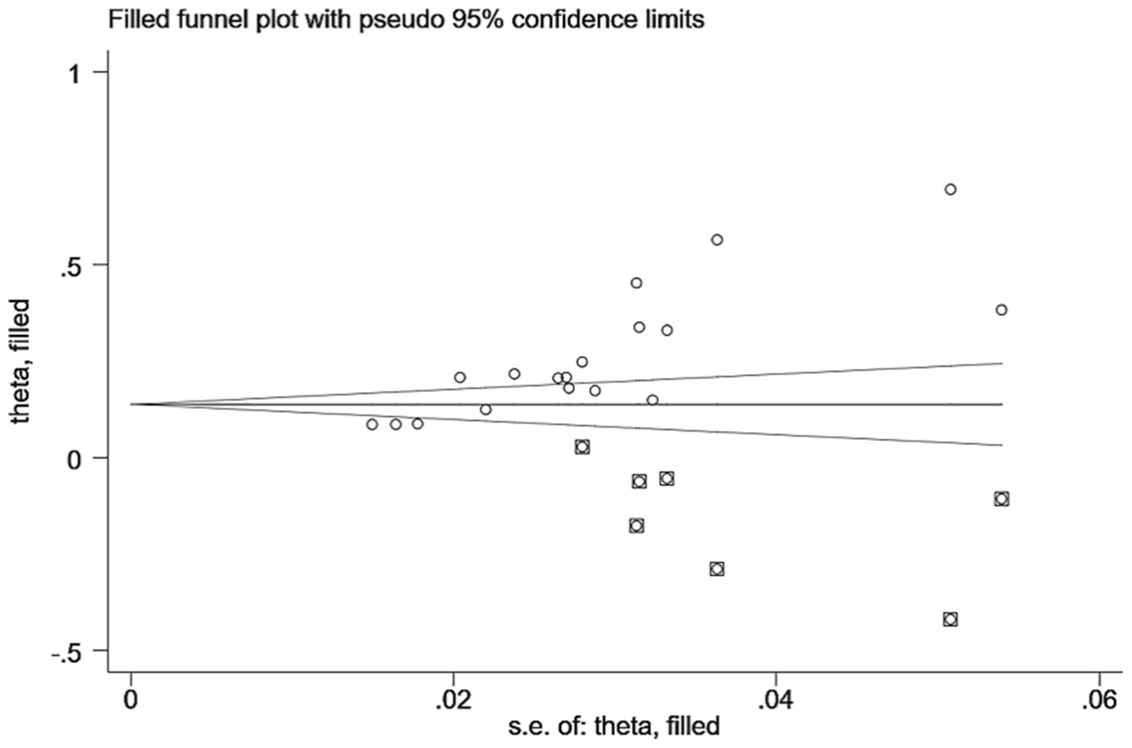
Results of the clipping method.

Sensitivity analysis showed no significant change in the combined prevalence of *CF* after excluding individual studies (Figure 3), indicating stable pooled prevalence results. The funnel shape of the *CF* prevalence study is essentially symmetrical (Figure 4). Based on the results of the Egger test (*p* < 0.001) and the Begg test (*p* = 0.001), it was suggested that the article may have some publication bias. Using the scissification method, it was determined that seven further studies would need to be included in this study to eliminate publication bias (Figure 5).

## 4. Discussion

Based on 18 studies (4,030 patients), the overall prevalence of *CF* in diabetic patients in China was 25.8%, and the prevalence of *CF* in diabetic patients ≥60 years old was 24.4%(the lower prevalence of *CF* in diabetic patients ≥60 years old may be related to the exclusion of Wang’s study (31) (the study could not extract data ≥60 years old)). The prevalence of *CF* varies in different countries and institutions according to the survey population and the assessment tool used. The general population prevalence of *CF* in other countries ranges from 1.0 to 8.9% (17, 20, 42–44), which is much higher in our study. The reasons may include a certain gap between China and developed countries in *per capita* medical resource occupancy. This generation of Chinese older adults individuals is more likely to engage in manual labor when they are young, have lower education level, are more prone to potential injuries and are less likely to use resources to maintain their health. Patients with diabetes are affected by diseases, and their physical health is more fragile and prone to unhealthy states.

In comparison with the prevalence of their disease *CF*: one study showed that the prevalence of *CF* in patients with chronic kidney disease was 15.2% (46), another Chinese study showed that the prevalence of *CF* in patients with hypertension was 9.8% (47), and this figure was 20.8% in patients with diabetes and hypertension (48), both of which were lower than the 25.8% prevalence of *CF* in diabetic patients in this study. In terms of more severe diseases, a survey of Korean patients with heart failure showed that the prevalence of *CF* in patients with heart failure was 34.5% (49), and another study mentioned that the prevalence of *CF* in older adults patients on dialysis was 35.9% (50), both of which were higher than the results of this study. This suggests that compared with more serious diseases, the physical health of patients with diabetes is relatively better, but compared with other long-term chronic diseases, the impact of diabetes on the occurrence of *CF* should be given more attention.

In the comparison of the prevalence of *CF* in patients with diabetes in the community, except for one study in India, which reached 21.8% (51), the prevalence of *CF* in other countries did not exceed 10% (52–55). The rate of 25.8% in our study was higher than that in other countries, but only India had a similar result. Both China and India have large populations, so this may be related to the *per capita* occupancy of health care resources. The results of this study suggest that the prevalence of *CF* in diabetic patients in China is high and that prevalence of *CF* in older adults diabetic patients is higher than that in the general older adults population. Therefore, it is necessary to improve the attention of medical staff and older adults diabetic patients.

Subgroup analyses showed that the prevalence varied by range. From the perspective of sample sources, the prevalence of *CF* in diabetic patients in Chinese communities is 25.8%, and the detection rate of *CF* in older adults hospitalized diabetic patients is 31.1%, indicating that the prevalence of *CF* in hospitals is higher than that in communities. The conclusion is the same as that of Liu et al. (56), which may be related to the poor health status of hospitalized patients compared with community patients. They are prone to negative emotions such as loneliness and depression, their daily activities in the hospital are limited, and their physical and mental work is reduced. In terms of sex, the prevalence of *CF* in women was higher than that in men, and the prevalence of *CF* in women was higher than that in men in both the community and hospital, consistent with the findings of Liu et al. (56). Whitson et al. (57) found that older women generally have more comorbidities than men, which may lead to a higher prevalence than men. In addition, vitamin D deficiency in older adults women after menopause may also be one of the causes. In terms of regional distribution, the detection rate of *CF* in southern patients was 21.6%, and the detection rate of *CF* in northern patients was 29.4%, indicating that the prevalence of southern patients was higher than that of northern patients, and the conclusion was the same as that of Liu et al. (56). The reason may be that the economic level of the south is relatively higher, the medical and health services are better, and diabetics patients can more easily access higher quality medical services and higher health standards. In terms of age distribution, the prevalence of *CF* in patients aged 60 ~ 69, 70 ~ 79, and ≥ 80 years increased gradually, suggesting that the prevalence of *CF* in patients with diabetes increases with age, which is consistent with previous studies (56, 58, 59). The reason for this may be that with increasing age, the physical function of the older adults gradually declines, accompanied by muscle loss, which promotes the occurrence and development of *CF.* The results suggest that there are differences in the prevalence of *CF* among diabetic patients in China in terms of sample source and regional distribution, and more longitudinal studies are needed to explore the reasons for the difference in prevalence between different regions. Targeted interventions need to be used for different regions and different patient groups in treatment.

This study showed that depression, malnutrition, age (≥70) (≥80), regular exercise, educational level (college or above), chronic disease ≥4 and high glycated hemoglobin (≥8.5) were the main factors related to *CF* in diabetic patients in China.

### 4.1. Depression

This study showed that depression was a protective factor for *CF* in diabetic patients in China. A study from Korea showed that patients’ depression was associated with *CF* (49). In addition, studies on patients with hypertension (47), chronic kidney disease (45), and dialysis (50) also showed the same conclusion, which may be related to the severity of the disease, high medical costs, and heavy burden of care, which make patients prone to pessimism. The mechanism of depression in *CF* may affect patients by slowing cognitive ability (60), reducing feeding and exercise initiative (61), and reducing social activities (62). At the same time, depression is also associated with the risk of adverse health outcomes such as suicide (63). Therefore, relevant medical personnel should put psychotherapy in the same important position as physical therapy and carry out psychotherapy under the guidance of professional psychologists.

### 4.2. Malnutrition

Malnutrition, according to the results of this study is a risk factor for diabetic patients with *CF.* This is in line with the findings of Chen et al. (50), a study of 340 maintenance hemodialysis patients, and may be related to the problems of metabolism and nutrient utilization in both diabetic and dialysis patients. Research has shown that insufficient nutrient intake is considered an important factor in physical frailty (64), while another study has shown that metabolic problems are also associated with cognitive decline in older people (64). For this, shall meet the needs of diabetes patients with *CF*, detailed in diabetes diet, nutrition, dietary intervention. At the same time, attention should be given to the potential malnutrition of low-income diabetic patients and corresponding interventions should be carried out to maintain their healthy nutritional status.

### 4.3. Age (≥70, ≥80)

This study shows that the age of diabetic patients in China is related to the prevalence of *CF.* This conclusion is consistent with previous studies (44, 45, 48, 50, 51), but age was not a relevant factor in a study of *CF* in hypertensive patients (47), which may be related to the fact that the population included in this study was mainly 60-79-years-old patients, and the age span was small. The study by Shimada et al. (65) showed that *CF* problems were more severe in patients older than 75 years. This suggests the need for interventions in accordance with age characteristics for patients of different ages. For example, older older adults patients may pay more attention to aerobic exercise and meditation, while younger older adults people may increase muscle strength and endurance training so that patients can obtain the maximum benefit from exercise therapy.

### 4.4. Regular exercise

The results of this study showed that regular exercise was a protective factor for *CF* in diabetic patients in China. Previous studies have suggested that exercise is not a protective factor for *CF* in hypertensive patients (47). The reason may be that diabetic patients have glucose utilization disorders, and they need to exercise regularly to stabilize postprandial blood glucose, reduce body weight, and improve insulin sensitivity, so they have higher exercise requirements than hypertensive patients. Other studies have concluded that physical exercises such as resistance exercise and aerobic exercise are beneficial for maintaining muscle strength, improving cognitive ability and reducing frailty in patients with diabetes (66–68). At the same time, studies have shown that long-term regular exercise is also conducive to relieving patients’ emotions and improving their quality of life. At the same time, studies have shown that muscle strength is related to the quality of life of the older adults individuals (69), and long-term regular exercise can also help the older adults individuals improve their quality of life (69, 70). People with diabetes are advised to exercise regularly to control blood sugar fluctuations. Diabetic patients with *CF* should carry out more targeted exercises that take into account both flexibility and coordination or add a certain proportion of cognitive training (such as meditation after exercise) to their daily exercise plan to take into account both physical and mental training.

### 4.5. High education level (college or above)

The results of this study suggest that a high level of education is a protective factor for *CF* in diabetic patients in China. This conclusion is consistent with previous studies (44, 50, 51), and another study showed that it is only an association factor but not an independent risk factor (45). The analysis may be related to the fact that people with higher education levels are more active in thinking, have stronger health concepts and are more likely to obtain disease-related knowledge and high-quality medical services. Although the education level of the individual is not a controllable factor, understanding the cultural level of the patients can help the relevant medical staff to carry out health education in a way that is easy for the patients to understand and formulate intervention measures in line with the patients’ educational level to improve their compliance with the intervention. Some studies have also built a risk prediction model by incorporating the patient’s cultural level into the model, which will help to predict the risk of *CF* in patients (71, 72).

### 4.6. Multimorbidity (chronic disease≥4)

Research shows that *CF* patients with diabetes risk factors include multimorbidity, in accordance with previous research results (47, 50). Kim et al. (73) showed a 6.419-fold increased risk of *CF* among community-dwelling older adults individuals with four or more chronic diseases. Multimorbidity can lead to a decline in physiological reserve, reduce the body’s resistance and tolerance, accelerate organ and system failure, and increase the potential risk of inappropriate medication in patients, leading to multiple factors acting on the body and increasing cognitive impairment and frailty (74). The treatment of older adults patients with multimorbidity needs to proceed from a systemic point of view and adopt the treatment modality that is most suitable for the patient’s condition.

### 4.7. High glycated hemoglobin (≥8.5)

High glycated hemoglobin is a risk factor for *CF* in diabetic patients in China, and a previous study on *CF* in diabetic patients with hypertension showed no correlation between high glycated hemoglobin and *CF*, but other laboratory indicators also showed no correlation with *CF* (48). The main reason is that the patient population is diabetic patients with hypertension, which is different from this study, and the survey area is a more developed area with better medical conditions.

Overall, we conducted a comprehensive search of the literature and maximized the collection of relevant studies to reduce selection bias. All study phases were independently assessed by two investigators, with discrepancies resolved through discussion. Data were pooled using a random-effects model to provide conservative estimates of the prevalence of *CF* in people with diabetes in China. Sensitivity and subgroup analyses were also performed to investigate possible causes of heterogeneity and to assess publication bias.

Our study still has some limitations. In our review, despite subgroup analyses of study participants, the source of heterogeneity was not identified, possibly from the study population and methodology. The differences in the population come from the differences in culture, diet, living habits, behavior patterns, etc. among the included groups. The differences in research methods mainly come from the differences in the combination of use scales. There is currently no optimal combination of the measurement scale for physical frailty and cognitive function, there are differences in the focus and scoring criteria between the scales used in each study, and there are different combinations and arrangements. At the same time, the included literature in this paper are all published literature, there may be some publication bias, and future studies need to include more high-quality literature to supplement the article conclusions.

## 5. Conclusion

In summary, the analysis results of this study showed that the prevalence of *CF* in Chinese diabetic patients was 25.8%, and the prevalence was high. Subgroup analysis showed that the prevalence was higher in hospitals than in the community, with women being more prevalent than men. With increasing age, course, and number of chronic diseases, the prevalence of *CF* gradually increases, and there are certain differences between the prevalence in different regions and cultural levels. Among the related factors, depression, malnutrition, advanced age, combined chronic diseases ≥4, and glycosylated hemoglobin ≥8.5 were risk factors for *CF.* A college education level or above education level and regular exercise are protective factors against *CF.* Future researchers should conduct standardized research on the assessment tools of *CF*, and medical workers should recognize the importance of *CF* in diabetic patients and actively take interventions to reduce the occurrence of diabetic *CF* and delay or prevent its progression.

## Author contributions

JP conducts major thesis writing, data extraction and data analysis, and article proofreading. LM conducts thesis writing, data extraction and partial data analysis. JW, YL, and SY provided ideas. QL provides ideas for papers and reviews articles. All authors contributed to the article and approved the submitted version.

## Funding

This investigation was supported by the Yunnan Province Natural Science Foundation Project (202001AU070117).
